# Syndecan-1 downregulates syndecan-4 expression by suppressing the ERK1/2 and p38 MAPK signaling pathways in cultured vascular endothelial cells

**DOI:** 10.1016/j.bbrep.2021.101001

**Published:** 2021-04-24

**Authors:** Takato Hara, Arisa Sato, Chika Yamamoto, Toshiyuki Kaji

**Affiliations:** a Faculty of Pharmaceutical Sciences, Toho University, Chiba, Japan; b Faculty of Pharmaceutical Sciences, Tokyo University of Science, Chiba, Japan

**Keywords:** Vascular endothelial cells, Syndecan-1, Syndecan-4, MAPK

## Abstract

Syndecan-1 and syndecan-4 are members of the syndecan family of transmembrane heparan sulfate proteoglycans. Vascular endothelial cells synthesize both species of proteoglycans and use them to regulate the blood coagulation-fibrinolytic system and their proliferation *via* their heparin-like activity and FGF-2 binding activity, respectively. However, little is known about the crosstalk between the expressions of the proteoglycan species. Previously, we reported that biglycan, a small leucine-rich dermatan sulfate proteoglycan, intensifies ALK5–Smad2/3 signaling by TGF-β_1_ and downregulates syndecan-4 expression in vascular endothelial cells. In the present study, we investigated the crosstalk between the expressions of syndecan-1 and other proteoglycan species (syndecan-4, perlecan, glypican-1, and biglycan) in bovine aortic endothelial cells in a culture system. These data suggested that syndecan-1 downregulated syndecan-4 expression by suppressing the endogenous FGF-2-dependent ERK1/2 pathway and FGF-2-independent p38 MAPK pathway in the cells. Moreover, this crosstalk was a one-way communication from syndecan-1 to syndecan-4, suggesting that syndecan-4 compensated for the reduced activity in the regulation of vascular endothelial cell functions caused by the decreased expression of syndecan-1 under certain conditions.

## Highlights

• Syndecan-1 downregulates syndecan-4 expression in vascular endothelial cells.
• The crosstalk is a one-way communication from syndecan-1 to syndecan-4.
• ERK1/2 pathway and p38 MAPK pathway are involved in the crosstalk.
• Suppression of ERK1/2 pathway by syndecan-1 is FGF-2-dependent.
• Suppression of p38 MAPK pathway by syndecan-1 is FGF-2-independent.

## Graphical abstract

**Figure:**
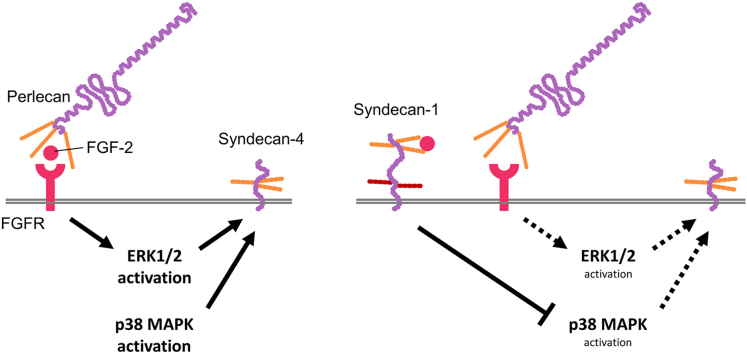

## 1 Introduction

Vascular endothelial cells cover the luminal surface of blood vessels in a monolayer and serve as a barrier between the blood and the subendothelial tissue. If the monolayer is repeatedly and severely damaged, platelets aggregate at the damaged site and release cytokines/growth factors such as platelet-derived growth factor [1] to promote vascular smooth muscle cell hyperplasia during atherosclerosis progression [2]. Fibroblast growth factor-2 (FGF-2) leakage from damaged endothelial cells promotes their proliferation [3] and the repair of damaged endothelial monolayers. It has been shown that FGF-2, FGF receptor (FGFR), and perlecan, a large heparan sulfate proteoglycan (HSPG), form a ternary complex [4] and induce signal transduction to stimulate proliferation.

Proteoglycans (PGs) are complex carbohydrates that consist of a core protein and one or more glycosaminoglycan side chains [5]. Vascular endothelial cells predominantly express cell membrane-associated HSPGs, including the large extracellular matrix HSPG perlecan [6], the syndecan family of transmembrane HSPGs [7,8], syndecan-1, syndecan-2, syndecan-3, syndecan-4, and glypican-1 [7]. The cells also express biglycan, a small leucine-rich dermatan sulfate PG (DSPG) [9]. HSPGs and DSPG can activate antithrombin III [7] and heparin cofactor II [10], respectively, and contribute to the anticoagulant properties of the vascular endothelium. Additionally, perlecan is reportedly responsible for binding FGF-2 to its receptor, whereas syndecans compete with perlecan in FGF-2-FGFR binding [11,12]. However, all HSPG species exhibit heparin-like anticoagulant activity [7].

Regulation of endothelial PG synthesis is important for understanding the mechanisms underlying the repair process of damaged endothelium and its anticoagulant properties. We revealed that FGF-2 [13] and transforming growth factor-β_1_ (TGF-β_1_) [14] regulate syndecan-4 synthesis *via* different intracellular signaling pathways. Specifically, FGF-2 upregulates endothelial syndecan-4 synthesis *via* the MEK1/2-ERK1/2 pathway in dense cells, whereas only transcriptional induction of syndecan-4 occurs at a low cell density *via* the Akt pathway. TGF-β_1_ also regulates syndecan-4 synthesis in a biphasic manner, and the Smad3–p38 MAPK and Smad2/3 pathways mediate early upregulation and late downregulation, respectively. Additionally, it was revealed that the hypoxia-inducible factor-1 pathway [15] and the p38 MAPK [16] pathway can mediate the upregulation of syndecan-4 expression, using organic-inorganic hybrid molecules as molecular probes.

Previously, we found that siRNA-mediated biglycan knockdown increases syndecan-4 expression *via* the Smad2/3 TGF-β signaling pathway in vascular endothelial cells [17]. This suggests that there may be crosstalk between the expression of different species of endothelial PGs. However, little is known about the crosstalk between expressions of the endothelial PGs. Since syndecan-1 and syndecan-4 appear to have similar functions in vascular endothelial cells, we hypothesized that syndecan-1 expression affects syndecan-4 expression in vascular endothelial cells. In the present study, we examined this hypothesis using a culture system of bovine aortic endothelial cells.

## 2 Materials and methods

### 2.1 Materials

Bovine aortic endothelial cells were obtained from Cell Applications (San Diego, CA, USA). Dulbecco's modified Eagle's medium (DMEM) was obtained from Nissui Pharmaceutical (Tokyo, Japan). Tissue culture dishes were obtained from Corning (Corning, NY, USA). Opti-MEM® Reduced Serum Medium, Lipofectamine® RNAiMAX Transfection Reagent, and a High-Capacity cDNA Reverse Transcription Kit were obtained from Thermo Fisher Scientific (Waltham, MA, USA). LY364947 and anti-GAPDH monoclonal antibody were obtained from Fujifilm Wako Pure Chemical Industries (Osaka, Japan). QIAzol Lysis Reagent was obtained from QIAGEN (Venlo, Netherlands). GeneAce SYBR® qPCR Mix α was obtained from Nippon Gene (Tokyo, Japan). Chondroitinase ABC and diethylaminoethyl-Sephacel (DEAE-Sephacel) were obtained from Sigma-Aldrich (St Louis, MO, USA). Heparinase II and heparinase III were purchased from IBEX Technologies (Quebec, Canada). Antibodies against syndecan-1 (sc-7099), syndecan-4 (sc-9497), and PD161570 were obtained from Santa Cruz Biotechnology (Santa Cruz, CA, USA). Anti-goat secondary antibody (ab6885) was obtained from Abcam (Bristol, UK). Antibodies against phospho-p44/42 MAPK (Erk1/2) (Thr202/Tyr204) (#9109), p44/42 MAPK (Erk1/2) (#9102), phosphor-p38 MAPK (#9211), p38 MAPK (#9212), phospho-SAPK/JNK (#9255), SAPK/JNK (#9252), anti-rabbit secondary antibody (#7074), and anti-mouse secondary antibody (#7076) were obtained from Cell Signaling Technology (Danvers, MA, USA). PD98059 and SB203580 were obtained from Cayman Chemical (Ann Arbor, MI, USA). Other reagents of the highest grade were obtained from Nacalai Tesque (Kyoto, Japan).

### 2.2 Cell culture and treatment

Bovine aortic endothelial cells, which have been widely used in PG research [[13], [14], [15], [16], [17], [18], [19], [20], [21]], at 23–30 passages were cultured in a humidified atmosphere of 5% CO_2_ at 37 °C in DMEM supplemented with 10% fetal bovine serum (FBS) in 35-mm dishes until 80% confluence. The cells were then incubated for 8 h at 37 °C in fresh DMEM supplemented with 10% FBS in the presence of the siRNA/Lipofectamine RNAiMAX mixture prepared as described previously [17]. The medium was changed to DMEM supplemented with 10% FBS and cells were incubated at 37 °C for 3, 6, 12, 16, and 24 h. The levels of syndecan-1 and syndecan-4 mRNAs and the expression of syndecan-4 core protein were determined by quantitative reverse transcription polymerase chain reaction (RT-PCR) and Western blot analysis, respectively. Separately, the cells were transfected with syndecan-1 siRNA for 8 h, treated with or without PD98059, SB203580, PD161570, or LY364947 for 1 h after changing the medium, and then incubated for 23 h in fresh DMEM supplemented with 10% FBS. Phosphorylation levels of MAPKs (ERK1/2, p38 MAPK, and JNK) were evaluated by Western blot analysis. The sequences of siRNAs for syndecan-1 (SDC-1-1 and SDC-1-2) and syndecan-4 (SDC-4-1 and SDC-4-2) are shown in Table S1.

### 2.3 PG core protein expression and Western blot analysis

PGs accumulated in the cell layer were extracted from the whole cell lysate, and the conditioned medium of vascular endothelial cells were concentrated as previously reported [15]. Concentrated PGs were digested with heparinase II/III in 100 mM Tris-HCl (pH 7.0) containing 10 mM calcium acetate and 18 mM sodium acetate (for syndecan-4 core protein) or with heparinase II/III and chondroitinase ABC (for syndecan-1 core protein) for 3 h at 37 °C. After digesting heparan sulfate and chondroitin sulfate chains with heparinase II/III and chondroitinase ABC, respectively, the PG core protein bands appeared by Western blot analysis, and the molecular mass of PG core proteins was determined using SDS-PAGE [15]. Western blot analysis was performed using samples obtained from multiple independent experiments to confirm reproducibility at least twice.

### 2.4 Quantitative RT-PCR analysis

Total RNA was extracted from vascular endothelial cells, and cDNA synthesis was performed as described previously [14]. Quantitative PCR was performed using GeneAce SYBR qPCR mix α with 1 ng cDNA and 0.2 μM primers (Table S2) in a StepOnePlus real-time PCR system (Applied Biosystems). The levels of syndecan-1, syndecan-4, biglycan, glypican-1, perlecan, FGF-2, and β_2_-microglobulin (B2M) transcripts were quantified using the relative standard curve method. The fold changes for each gene were normalized to the intensity value of B2M.

### 2.5 Statistical analysis

Data were analyzed for statistical significance by analysis of variance and Welch's *t*-test, as appropriate. *P* values less than 0.05 were considered to indicate statistically significant differences.

## 3 Results

### 3.1 Suppression of syndecan-1 expression induces syndecan-4 expression

**Fig. 1:**
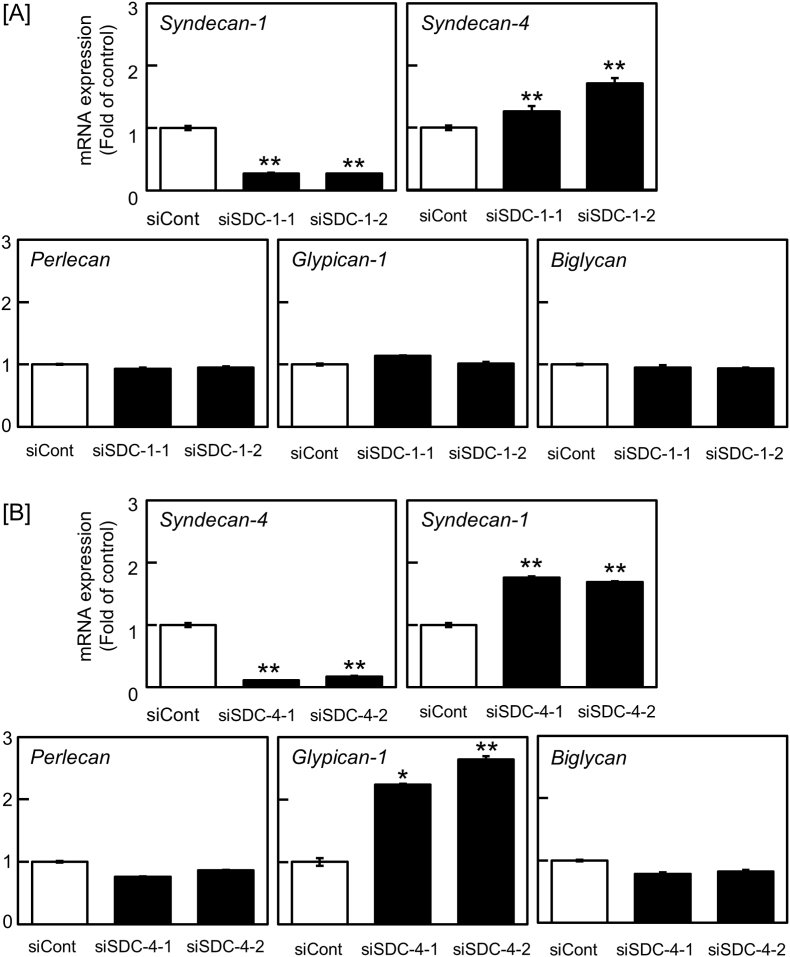
Effects of siRNA-mediated syndecan-1 or syndecan-4 knockdown on the levels of PG mRNAs in vascular endothelial cells. Bovine aortic endothelial cells were transfected with siCont, siSDC-1-1, siSDC-1-2, siSDC-4-1, or siSDC-4-2 for 8 h, and then incubated for 16 h in fresh medium. [A] Effects of siRNA-mediated syndecan-1 knockdown on the levels of other PG mRNAs. [B] Effects of siRNA-mediated syndecan-4 knockdown on the levels of other PG mRNAs. Values are the means ± SE of three independent experiments. Significantly different from the corresponding “siCont”, *P < 0.05; **P < 0.01.

**Fig. 2:**
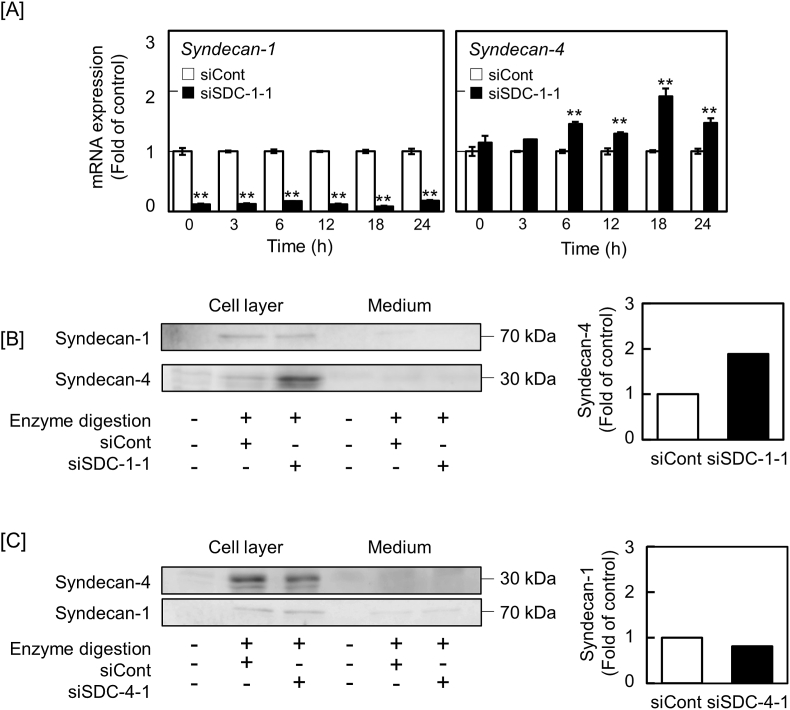
Effects of siRNA-mediated syndecan-1 knockdown on syndecan-1 mRNA and core protein expression, and the effects of siRNA-mediated syndecan-4 knockdown on syndecan-1 core protein expression in vascular endothelial cells. [A] Time course of the levels of syndecan-4 mRNA after syndecan-1 knockdown. Bovine aortic endothelial cells were transfected with siCont or siSDC-1-1 for 8 h, and then incubated for 3, 6, 12, 18, and 24 h in fresh medium. Values are the means ± SE of three independent experiments. **Significantly different from the corresponding siCont, *P* < 0.01. [B] Expression of syndecan-4 core protein after syndecan-1 knockdown. Bovine aortic endothelial cells were transfected with siCont or siSDC-1-1 for 8 h, and then incubated for 24 h in fresh medium. The blots present syndecan-1 and syndecan-4 expression (left panels) and the measured proportion of the intensity of syndecan-4 expression in the cell layer (right panel). [C] Expression of syndecan-1 core protein after syndecan-4 knockdown. Bovine aortic endothelial cells were transfected with siCont or siSDC-4-1 for 8 h, and then incubated for 24 h in fresh medium. The blots present syndecan-1 and syndecan-4 expression (left panels) and the measured proportion of the intensity of syndecan-1 expression in the cell layer (right panel).

Fig. 1A shows the effect of siRNA-mediated syndecan-1 knockdown on the expression of mRNAs coding for other species of PGs in bovine aortic endothelial cells. Suppression of syndecan-1 mRNA expression by siSDC-1-1 and siSDC-1-2 resulted in elevation of syndecan-4 mRNA levels by 1.26-fold and 1.72-fold, respectively. Perlecan, glypican-1, and biglycan mRNA levels were unaffected by syndecan-1 knockdown. The expression levels of syndecan-2 and syndecan-3 were not analyzed in this study because we have previously shown that their expression levels are very low in vascular endothelial cells [17]. On the other hand, as shown in Fig. 1B, syndecan-4 knockdown by siSDC-4-1 and siSDC-4-2 significantly elevated the levels of syndecan-1 by 1.76-fold and 1.69-fold and glypican-1 by 2.24-fold and 2.64-fold, respectively, without changes in the levels of perlecan and biglycan mRNAs. A time-course study indicated that syndecan-4 mRNA levels were elevated by syndecan-1 knockdown after 6 h or more (Fig. 2A), and increased syndecan-4 core protein expression was confirmed by Western blot analysis. The core protein bands of syndecan-1 and syndecan-4 were detected only in the cell layer (Fig. 2B), because these PGs are transmembrane proteins. However, syndecan-4 knockdown did not change the expression of syndecan-1 core protein (Fig. 2C); the core protein of glypican-1 was not detected. Taken together, it is suggested that there is a crosstalk between the expression of syndecan-1 and syndecan-4, and that this is a one-way communication from syndecan-1 to syndecan-4 in vascular endothelial cells.

### 3.2 ERK1/2 and p38 MAPK pathways involved in induction of syndecan-4 expression by syndecan-1 suppression

**Fig. 3:**
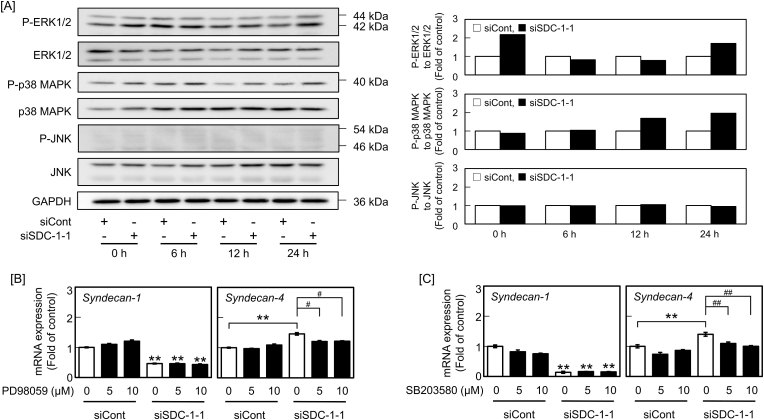
Activation of MAPKs (ERK1/2, p38 MAPK, and JNK) after siRNA-mediated syndecan-1 knockdown and the involvement of this activation in the elevation of syndecan-4 mRNA level in vascular endothelial cells. [A] Activation of MAPKs after siRNA-mediated syndecan-1 knockdown. Bovine aortic endothelial cells were transfected with siCont or siSDC-1-1 for 8 h and incubated for 6, 12, and 24 h in fresh medium. The blots present MAPK expression (left panels) and the calculated ratio of the intensity of phosphorylated MAPK expression to that of total MAPK expression (right panels). The values presented in the bar graphs were calculated using data presented in the blots. [B,C] Involvement of ERK1/2 and p38 MAPK activation in the elevation of the syndecan-4 mRNA level by siRNA-mediated syndecan-1 knockdown. Bovine aortic endothelial cells were transfected with siCont or siSDC-1-1 for 8 h; treated with [B] PD98059 and [C] SB203580 at 5 and 10 μM for 1 h after changing medium; and incubated for 23 h in fresh medium. Values are the means ± SE of three independent experiments. Significantly different from the corresponding siCont, **P < 0.01. ^##^Significantly different from the corresponding “absence of inhibitor”, ^#^*P* < 0.05; ^##^*P* < 0.01.

We recently reported that p38 MAPK induces syndecan-4 expression in aortic endothelial cells [14,16]. To examine the involvement of MAPKs (ERK1/2, p38 MAPK, and JNK) in the elevation of syndecan-4 mRNA levels by siRNA-mediated syndecan-1 knockdown, the phosphorylation of MAPKs in vascular endothelial cells was examined after syndecan-1 knockdown. The phosphorylation of ERK1/2 and p38 MAPK was elevated by syndecan-1 knockdown in a time-dependent manner, suggesting that the ERK1/2 and p38 MAPK pathways were activated by the suppression of syndecan-1 expression in vascular endothelial cells. Additionally, elevation of syndecan-4 mRNA levels by syndecan-1 knockdown was significantly suppressed by the MEK1/2 inhibitor PD98059 (Fig. 3B) and p38 MAPK inhibitor SB203580 (Fig. 3C) without any morphological changes in cells, suggesting that the ERK1/2 and p38 MAPK pathways mediate the induction of syndecan-4 expression in syndecan-1-suppressed vascular endothelial cells.

### 3.3 FGFR-mediated ERK1/2 activation is involved in syndecan-4 induction by syndecan-1 suppression

FGF-2 and TGF-β_1_ are important regulators of vascular endothelial cell functions such as proliferation [22,23], FGF-2-induced sustained phosphorylation of ERK1/2 [24], and syndecan-4 expression [13]. TGF-β_1_ also activates p38 MAPK and induces syndecan-4 expression in vascular endothelial cells [14]. Therefore, we next analyzed whether FGFR and ALK5, a functional receptor for FGF-2 and TGF-β_1_, respectively, are involved in the syndecan-1 knockdown-mediated activation of ERK1/2 and p38 MAPK.

**Fig. 4:**
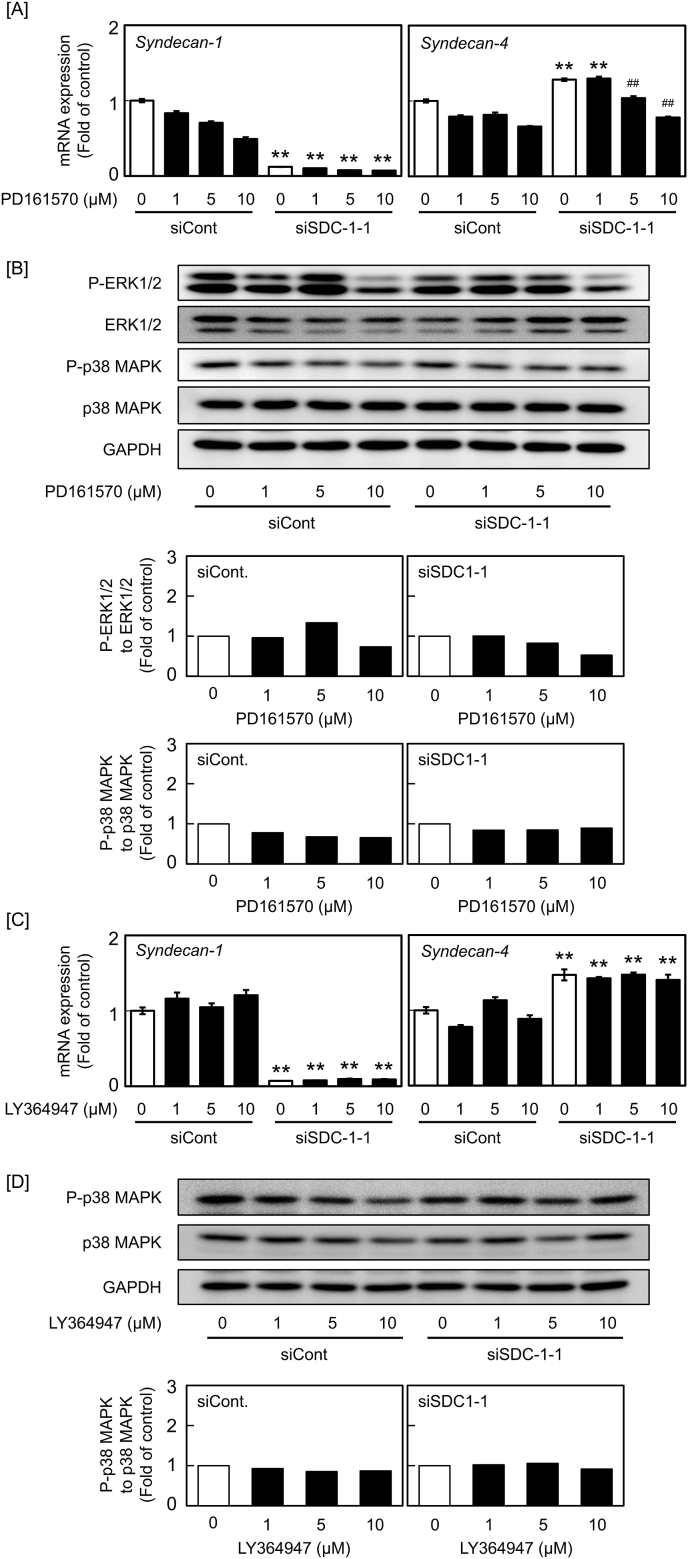
Involvement of FGFR and ALK5 in the elevation of the syndecan-4 mRNA level by siRNA-mediated syndecan-1 knockdown in vascular endothelial cells. Bovine aortic endothelial cells were transfected with siCont or siSDC-1-1 for 8 h; treated with [A] PD161570 and [C] LY364947 at 1, 5, and 10 μM for 1 h; and incubated for 23 h in fresh medium. Values are the means ± SE of three independent experiments. **Significantly different from the corresponding siCont, *P* < 0.01. ^##^Significantly different from the corresponding “absence of inhibitor”, *P* < 0.01. [B] Involvement of FGFR in the elevation of ERK1/2 and p38 MAPK phosphorylation by siRNA-mediated syndecan-1 knockdown. Bovine aortic endothelial cells were transfected with siCont or siSDC-1-1 for 8 h; treated with PD161570 at 1, 5, and 10 μM for 1 h; and incubated for 23 h in fresh medium. The blots present ERK1/2 and p38 MAPK expression (top panels) and the calculated ratio of the intensity of phosphorylated ERK1/2 (middle panels) and phosphorylated p38 MAPK (bottom panels) expressions to that of total ERK1/2 and p38 MAPK expressions, respectively. The values presented in the bar graphs were calculated using data presented in the blots. [D] Involvement of ALK5 in the elevation of p38 MAPK phosphorylation by siRNA-mediated syndecan-1 knockdown. Bovine aortic endothelial cells were transfected with siCont or siSDC-1-1 for 8 h; treated with LY364947 at 1, 5, and 10 μM for 1 h; and incubated for 23 h in fresh medium. The blots present p38 MAPK expression (top panels) and the calculated ratio of the intensity of phosphorylated p38 MAPK expression (bottom panels) to that of total p38 MAPK expression. The values presented in the bar graphs were calculated using data presented in the blots.

We have previously reported that FGF-2 slightly induces syndecan-1 expression [13], and the FGFR blocker PD161570 alone suppresses syndecan-1 mRNA expression without any morphological changes in cells (Fig. 4A). This suggests that endogenous FGF-2 is involved in syndecan-1 expression induction in vascular endothelial cells. Consequently, when syndecan-1 mRNA levels were suppressed by siRNA in the presence of the FGFR blocker, the elevation of the syndecan-4 mRNA level was significantly reduced (Fig. 4A). In this experiment, activation of ERK1/2 and p38 MAPK by transfecting cells with siSDC1-1 (Fig. 3A) was not observed, probably because of the different experimental conditions. PD161570 (10 μM) reduced the phosphorylation of ERK1/2 in the presence or absence of syndecan-1 siRNA. In contrast, p38 MAPK phosphorylation was hardly affected by the FGFR blocker (Fig. 4B). Although syndecan-1 knockdown resulted in elevation of syndecan-4 mRNA levels, the ALK5 blocker LY364947 did not influence this elevation (Fig. 4C). Additionally, p38 MAPK phosphorylation was unaffected by the ALK5 blocker (Fig. 4D).

## 4 Discussion

Syndecan-1 and syndecan-4 are members of the syndecan family of small transmembrane HSPGs; syndecan-1 has chondroitin sulfate chains as well as HS chains, while syndecan-4 has only HS chains [25]. Although the functions of syndecans depend on the cell type, it appears that the roles of syndecan-1 and syndecan-4 are similar in vascular endothelial cells. For example, both syndecan-1 and syndecan-4 have antithrombin III activating activity [7,8] and contribute to the anticoagulant properties of vascular endothelial cell layers. When vascular endothelial cells are injured, FGF-2 leaks from the damaged cells [22] and promotes the migration and proliferation of cells near the damaged site to repair the damaged cell layer [3]. Both syndecan-1 and syndecan-4 compete with perlecan in FGF-2-FGFR binding [11,12]; the syndecans may negatively regulate FGF-2 activity in vascular endothelial cells. It is possible that syndecan-1 and syndecan-4 regulate cell functions by different mechanisms [[26], [27], [28]]. In the present study, we demonstrated that siRNA-mediated syndecan-1 knockdown increased syndecan-4 expression in vascular endothelial cells. In other words, it is suggested that endothelial syndecan-1 downregulates endothelial syndecan-4 expression. Additionally, this crosstalk was a one-way communication from syndecan-1 to syndecan-4, suggesting that syndecan-4 compensates for the lowered regulation of vascular endothelial cell functions mediated by FGF-2 and reduced antithrombin III activation caused by decreased syndecan-1; however, the opposite would not occur.

In a previous study, the hypoxia-inducible factor-1α/β pathway activated by 1,10-phenanthroline [15], the p38 MAPK pathway activated by copper(II) bis(diethyldithiocarbamate) [16], and the ERK1/2 pathway activated by FGF-2 [13] upregulate syndecan-4 expression in vascular endothelial cells. Additionally, the Smad3-p38 MAPK pathway activated by TGF-β_1_ also elevates endothelial syndecan-4 expression [14]. It is postulated that the MAPKs (ERK1/2, p38 MAPK, and JNK) may be involved in the upregulation of syndecan-4 expression by syndecan-1 knockdown. The present data indicate that the ERK1/2 and p38 MAPK pathways mediate this upregulation, and the ERK1/2 pathway is activated downstream of FGFR, but not downstream of ALK5. It is suggested that the activity of endogenous FGF-2 is elevated when syndecan-1 expression is decreased. Endogenous FGF-2 forms a ternary complex with FGFR and a large HSPG perlecan [4] and activates the ERK1/2 pathway as a downstream pathway [13]. It is likely that syndecan-1 competitively acts on perlecan, inhibits ternary complex formation, and reduces ERK1/2 pathway activation, resulting in suppression of syndecan-4 expression. Because syndecan-4 and syndecan-1 can compete with perlecan in the binding of FGF-2 to FGFR, it is likely that siRNA-mediated syndecan-4 knockdown would induce syndecan-1 expression, but this crosstalk is one-way from syndecan-1 to syndecan-4. In our previous study, we showed that FGF-2 highly elevated the syndecan-4 transcript, but not that of syndecan-1 [13]. This may explain why one-way traffic occurs. On the other hand, the mechanisms underlying the activation of p38 MAPK by syndecan-1 knockdown remain to be elucidated, although it has been shown that this activation is independent of FGFR and ALK5.

It has been reported that syndecan-1 and syndecan-4 are involved in the progression of vascular lesions. Arteriosclerosis is a vascular lesion characterized by neointimal hyperplasia of the vascular wall. In the atherosclerotic vascular wall, excess proliferation of vascular smooth muscle cells as well as lipid accumulation is observed. Syndecan-1 inhibits vascular smooth muscle cell proliferation [29], whereas syndecan-4 positively affects proliferation [30]; syndecan-4 is required for the migration and proliferation of vascular smooth muscle cells stimulated by thrombin [31]. Additionally, neointimal vascular smooth muscle cells highly express both syndecan-1 and syndecan-4 [32,33]. Regarding PGs, it is postulated that their expression in atherosclerosis depends on the balance between the expressions of syndecan-1 and syndecan-4. For example, if the increase in the expression of syndecan-1 is lower than that in the expression of syndecan-4 in neointimal smooth muscle cells, atherosclerosis progression influenced by the imbalance between the expressions of syndecan-1-syndecan-4 can be accelerated. Crosstalk occurring between syndecan-1 and syndecan-4 expressions, i.e., upregulation of syndecan-4 expression by downregulation of syndecan-1 expression under certain conditions in atherosclerosis may be one of the factors responsible for atherosclerosis progression.

Experimentally, it was shown that siRNA-mediated syndecan-1 knockdown induced syndecan-4 expression in vascular endothelial cells. This indicated that the induction was mediated by the ERK1/2 and p38 MAPK pathways. The ERK1/2 pathway was activated downstream of FGFR, while the mechanisms underlying p38 MAPK pathway activation were independent of FGFR and ALK5. Additionally, it was revealed that the crosstalk between syndecan-1 and syndecan-4 expression is a one-way communication from syndecan-1 to syndecan-4. This may be due to the inability of FGF-2 to induce syndecan-1 expression. Taken together, these results suggest that syndecan-1 suppresses the activation of both the ERK1/2 and p38 MAPK pathways and reduces syndecan-4 expression in vascular endothelial cells. Physiologically, syndecan-4 may compensate for the lowered regulation of vascular endothelial cell functions caused by decreased syndecan-1; however, the opposite would not occur. Further studies should be performed to examine whether there is crosstalk regulation among PG species, as we observed between biglycan and syndecan-4 [17] in the previous study, and between syndecan-1 and syndecan-4 in the present study, in vascular endothelial cells.
