# Supplementary material for: Syndecan-1 downregulates syndecan-4 expression by suppressing the ERK1/2 and p38 MAPK signaling pathways in cultured vascular endothelial cells
Source: Biochem Biophys Rep. 2021 Apr 24;26:101001. doi: 10.1016/j.bbrep.2021.101001 (PMC8099740; doi:10.1016/j.bbrep.2021.101001)
Supplement: Multimedia component 1 [file mmc1.docx]

**Table S1.** The sequences of sense and antisense strands of siRNA

| Gene | Sense (5’→3’) | Antisense (3’→5’) |
| --- | --- | --- |
| SDC-1-1 | AAACUAACCUAGACAUUGGdTdT | CCAAUGUCUAGGUUAGUUUdTdT |
| SDC-1-2 | UUGGAAUAUUCCUGAUUCCdTdT | GGAAUCAGGAAUAUUCCAAdTdT |
| SDC-4-1 | UUGUAGAUGGGUUUCUUGCdTdT | GCAAGAAACCCAUCUACAAdTdT |
| SDC-4-2 | AUUAUAUCUCCAACUCUUGdTdT | CAAGAGUUGGAGAUAUAAUdTdT |
| Negative control (siCont) | UUCUCCGAACGUGUCACGUdTdT | ACGUGACACGUUCGGAGAAdTdT |
