# Supplementary material for: Syndecan-1 downregulates syndecan-4 expression by suppressing the ERK1/2 and p38 MAPK signaling pathways in cultured vascular endothelial cells
Source: Biochem Biophys Rep. 2021 Apr 24;26:101001. doi: 10.1016/j.bbrep.2021.101001 (PMC8099740; doi:10.1016/j.bbrep.2021.101001)
Supplement: Multimedia component 2 [file mmc2.docx]

**Table S2**. Bovine gene-specific primers for quantitative RT-PCR

| Gene | Sense (5’→3’) | Antisense (3’→5’) |
| --- | --- | --- |
| Syndecan-1 | CAGTCAGGAGACAGCATCAG | CCGACAGACATTCCATACC |
| Syndecan-4 | TTGCCGTCTTCCTCGTGC | AGGCGTAGAACTCATTGGTGG |
| Biglycan | GCTGCCACTGCCATCTGAG | CGAGGACCAAGGCGTAG |
| Glypican-1 | GAAGGTCGGCAGGAAGAG | CCAGGAGCAGCAGAGGA |
| Perlecan | GCTGAGGGCGTACGATGG | TGCCCAGGCTCGGAACT |
| FGF-2 | AACCGTTACCTTGCTATG | CCCAGTTCGTTTCAGTGCC |
| B2M | CCATCCAGCGTCCTCCAAAGA | TTCAATCTGGGGTGGATGGAA |
